# Analysis of Publically Available Skin Sensitization Data from REACH Registrations 2008–2014

**DOI:** 10.14573/altex.1510055

**Published:** 2016-02-11

**Authors:** Thomas Luechtefeld, Alexandra Maertens, Daniel P. Russo, Costanza Rovida, Hao Zhu, Thomas Hartung

**Affiliations:** 1 Center for Alternatives to Animal Testing (CAAT), Johns Hopkins Bloomberg School of Public Health, Environmental Health Sciences, Baltimore, MD, USA; 2 The Rutgers Center for Computational & Integrative Biology, Rutgers University at Camden, NJ, USA; 3 Department of Chemistry, Rutgers University at Camden, NJ, USA; 4 CAAT-Europe, University of Konstanz, Konstanz, Germany

**Keywords:** animal testing alternatives, allergic contact dermatitis, *in silico*, chemical safety, computational toxicology

## Abstract

The public data on skin sensitization from REACH registrations already included 19,111 studies on skin sensitization in December 2014, making it the largest repository of such data so far (1,470 substances with mouse LLNA, 2,787 with GPMT, 762 with both *in vivo* and *in vitro* and 139 with only *in vitro* data). 21% were classified as sensitizers. The extracted skin sensitization data was analyzed to identify relationships in skin sensitization guidelines, visualize structural relationships of sensitizers, and build models to predict sensitization.

A chemical with molecular weight > 500 Da is generally considered non-sensitizing owing to low bioavailability, but 49 sensitizing chemicals with a molecular weight > 500 Da were found.

A chemical similarity map was produced using PubChem’s 2D Tanimoto similarity metric and Gephi force layout visualization. Nine clusters of chemicals were identified by Blondel’s module recognition algorithm revealing wide module-dependent variation.

Approximately 31% of mapped chemicals are Michael’s acceptors but alone this does not imply skin sensitization. A simple sensitization model using molecular weight and five ToxTree structural alerts showed a balanced accuracy of 65.8% (specificity 80.4%, sensitivity 51.4%), demonstrating that structural alerts have information value.

A simple variant of k-nearest neighbors outperformed the ToxTree approach even at 75% similarity threshold (82% balanced accuracy at 0.95 threshold). At higher thresholds, the balanced accuracy increased. Lower similarity thresholds decrease sensitivity faster than specificity.

This analysis scopes the landscape of chemical skin sensitization, demonstrating the value of large public datasets for health hazard prediction.

## 1 Introduction

While computational toxicology has recently seen the collection of several large-scale datasets (e.g., US EPA’s ToxCast, the Tox21 alliance of US agencies), the data collected by REACH (Regulation (EC) 1907/2006), owing to its legislative nature as a central repository for testing data, is the largest collection of toxicology data today relating to *in vitro* and *in vivo* studies. However, REACH dossiers submitted to the European Chemicals Agency (ECHA) are currently not in a machine readable format and any workflows involving the public summary data in REACH depend on a slow and error-prone process of manual extraction.

Here, we seek to demonstrate the extent and diversity of the public REACH dataset – a dataset that far surpasses most existing datasets used for computational toxicology – and show, as a case study, how an open-access REACH program would allow profound change in the analysis of skin sensitization by chemical substances. Using REACH data, we were able to find *in vivo* skin sensitization data for thousands of chemicals. To leverage this dataset we additionally rely on structure data and incorporate reaction alert data.

Skin sensitization provides a good case study to demonstrate the value of REACH data to computational toxicology. REACH requires skin sensitization data for all chemicals produced or marketed in Europe at a volume of 1 ton per year or more, the threshold of the legislation (REACH Annex VII, REACH Annex VIII; Hartung, 2010). This allows the creation of a well populated chemical similarity map. Already at the time point of this study, 7,697 substances with sensitization studies were available in the database with several tens of thousands expected by 2018. We first used ToxTree (Enoch et al., 2007) to develop a simple heuristic for skin sensitization prediction (modeling skin sensitization as a two class sensitizer/non-sensitizer problem) and evaluated its strength relative to a simple k-nearest neighbors (KNN) variant (Altman, 1992).

Skin sensitization, which clinically manifests in humans as allergic contact dermatitis (ACD), is an increasingly common concern among both regulators and the general public. Recent data indicate that an estimated 15–20% of the general population suffers from contact allergy (Thyssen et al., 2007). US Bureau of Labor Statistics (BLS) data shows that occupational skin diseases currently account for 10–15% of all occupational illness^1^. It has been estimated that the true number of occupational skin diseases may be in the order of 10–50 times higher than reported by the BLS (Lushniak, 2004). This would potentially raise the number of occupational skin disease cases in the US to between 400,000 and 2 million per year. Occupational contact dermatitis is particularly prevalent in the personal services industry, with an estimated prevalence of 1.2% percent, e.g., in the beauty/haircare industry (Warshaw et al., 2012), as well as high prevalences in the petrochemical, rubber, plastic, metal and automotive industries (McDonald et al., 2006).

Many chemicals used in occupational settings have not yet been tested for skin sensitization potential. Noteworthy, only one in eight premarketing notifications to EPA is submitted with any toxicological data. Although the replacement of the guinea pig maximization test (GPMT) with the mouse local lymph node assay (LLNA) allows for a reduction in animal use as well as suffering, there is still a strong need for an *in vitro* or *in silico* replacement (Adler et al., 2011; Basketter et al., 2012; Leist et al., 2014; Reisinger et al., 2015). The LLNA is prescribed by REACH for new test data, but the legislation allows the use of existing guinea pig or other data. Currently, no alternative approach has been formally accepted by ECHA for skin sensitization, but the legislation allows the use of alternative methods in weight-of-evidence approaches (Linkov et al., 2015). Integrated testing strategies are under development (Jaworska et al., 2011; ECVAM, 2013; Rovida et al., 2015).

Sensitization prediction has been approached in many (Q) SARs (Jaworska et al., 2013; Hirota et al., 2013; Luechtefeld et al., 2015). (Q)SARs typically have a limited applicability domain and are often over-fitted to the dataset from which they were derived (Hartung and Hoffmann, 2009). Supervised learning methods typically draw from *in vitro* data, with recent advances made in Jaworska et al.’s Bayesian network approach, Hirota et al.’s Artificial Neural Network and more (Jaworska et al., 2013; Hirota et al., 2013; Pirone et al., 2014; Luechtefeld et al., 2015). *In vitro* data allows direct measurement of features in OECD adverse outcome pathways, such as cysteine reactivity and dendritic cell activation, and as such provide a strong feature set for sensitization prediction. However, many of the *in vitro* features used in these publications have been measured for only a small subset of the chemical universe.

## 2 Methods

Multiple programming languages, packages and database tools were used in the development of this project, including SCALA, Java, Python, MongoDB, SQL, HTML Unit and Gephi. For details see Luechtefeld et al. (2016, this issue).

### 2.1 ECHA downloads for REACH dossiers

Data was downloaded from ECHA using HTMLUnit, an open source Java “Guiless browser” library (Bowler, 2002). Implementation of ECHA dossier download automation used the functional programming language SCALA (Odersky et al., 2004). In December 2014, a total of 10,588 dossiers for 9,801 chemicals were retrieved.

### 2.2 MongoDB

A MongoDB database^2^ was generated from the REACH data (Chodorow, 2013). The database was generated by automated data extraction from ECHA dossier URLs via the SCALA driver ReactiveMongo (Godbillon, 2015).

### 2.3 PubChem

PubChem’s Power User Gateway provided data on chemical similarity, chemical properties including molecular weight, and chemical identification information (common names, SMILES, etc.) (Bolton et al., 2008; Steinbeck et al., 2003).

### 2.4 Gephi

Gephi, a network visualization tool, was used to construct and analyze similarity networks (Bastian et al., 2009). The code for Gephi is available^4^. Custom code was written to export chemical similarity from MongoDB into a Gephi-readable format.

### 2.5 Force layout

The force atlas algorithm (Jacomy et al., 2014) was used for generation of chemical similarity networks. The force atlas algorithm is a physical simulation where graph nodes are treated as charged particles that repel each other and edges are treated as springs causing their associated nodes to be attracted. The following parameters were used for generation of the layout:

Inertia: 0.1, Repulsion: 200.0, Attraction: 10.0, Maximum displacement: 10.0, Auto stabilize: True, Autostabilize Strength: 80.0, Autostabilize sensibility: 0.2, Gravity: 9000, Attraction Distribution: false, Adjust by Sizes: True, Speed: 10.0

### 2.6 Chemical similarity

Construction of a chemical similarity map was done using PubChem’s 2D conformational substructure vectors as accessed through the Chemical Development Kit (CDK)^4^ (Steinbeck et al., 2003). Chemical similarity was calculated using Tanimoto distance, which is calculated by the number of shared substructures between chemicals divided by the total number of substructures in both chemicals and takes on a value between 1 (perfect similarity) and 0 (no similarity).

Using chemical similarity numbers, a chemical similarity map can be constructed and laid out using the force layout algorithm. This map is defined by the substances (called nodes in graph parlance) and edges (similarity between nodes).

K-Core filtration was used to filter out non-central substances in the chemical similarity map. K-Core filtration iteratively removes nodes (chemicals here) with the fewest neighbors first until all nodes have at least k neighbors. The result is a more cohesive similarity network at the cost of losing some interesting subsets of the network. K-Core filtration has been used successfully in discovering useful network structures in protein-protein networks (Altaf-Ul-Amine et al., 2003; Wuchty and Almaas, 2005). The Blondel et al. module recognition algorithm (Blondel et al., 2008) is used to identify clusters of chemicals. More detailed explanation of the construction and analysis of chemical modules is given in Luechtefeld et al. (2016, this issue).

Visualizing sensitization in the chemical similarity map requires substance sensitization status labeling. Chemical sensitization status is set to positive if there exists any “experimental key skin sensitization” study with a positive result for the given chemical or if there is classification and labeling data identifying the chemical as positive for H317.

Chemical similarity enables the use of KNN models for chemical classification. Here we implement a simple variant of KNN as a proof of principle for classification by similarity given large datasets (Altman, 1992).

Our similarity-based classification method is implemented as follows:

1. Choose minimum similarity parameter *T*.
2. Let *A_ij_* be the similarity between chemicals *i* and *j.*
3. Let *N_i_* be the set of chemicals with similarity *Aij* > *T*.
4. Predict sensitizer if majority of neighbors are sensitizers.
5. Predict non-sensitizer if majority neighbors are non-sensitizers.
6. In the event of a tie, predict sensitizer.

This instance-based learning approach is compared with a simple heuristic model of skin sensitization using molecular weight and structural alerts.

### 2.7 Module entropy

To identify which substructures provide the most information for sensitization status we used entropy calculations. Entropy is a metric with values between 0 and 1 and is higher for modules with balanced mixtures of sensitizers and non-sensitizers. Entropy is low for modules with imbalanced mixtures. A module consisting of all sensitizers would have 0 entropy.

Equation 1 Entropy equation for a module M; sens = sensitizer, nonSens = non-sensitizer

H(M)=-P(sens)∗log(P(sens))-p(nonsens)∗log(P(nonsens))

When a structural alert successfully divides a module into sensitizers and non-sensitizers, it is said to provide high information gain. This information gain is found quantitatively by evaluating entropy on 3 groups. The parent group (a substance module) and two child groups (substances in the module that are positive for a structural alert vs. those that are negative). The entropy for each subgroup is found. The difference between subgroup entropy (averaged by size of subgroup) and parent entropy is the information gain.

Equation 2 Structural alert information gain is the entropy difference of the parent group *H_p_* and the entropy of groups positive and negative for the given alert *H_pa_*, *H_pn_* (multiplied by the probability of the alert)

$$I(M|\mathrm{alert})=H_{p}-p(\mathrm{alert})H_{pa}-p(!\mathrm{alert})H_{pn}$$

As an example, if a structural alert divides a module’s substances into 2 subgroups, one with all sensitizers and one with no sensitizers, then the child groups both have entropy of 0 and thus the information gain is complete *(I(M|alert) = H(M)).* Since the parent group’s entropy is greater than 0, the information gain is equal to the entropy in the parent group and thus no more information can be gathered.

## 3 Results

### 3.1 Sensitization data exists in greater abundance in the REACH dataset than in other existing datasets

REACH requires assessment of skin sensitization potential for all registered substances (REACH Annex VII, REACH Annex VIII). Even in case of data-waiving for skin sensitization, which REACH allows under certain circumstances, substance dossiers collect many studies related to sensitization. The large REACH dataset, though biased by the high-production volume chemicals registered in 2010 and 2013, allowed assessment of the prevalence of skin sensitizers among industrial chemicals, critical information for the construction of any testing strategy (Hoffmann et al., 2005; Hartung et al., 2013; Rovida et al., 2015).

**Fig. 1:**
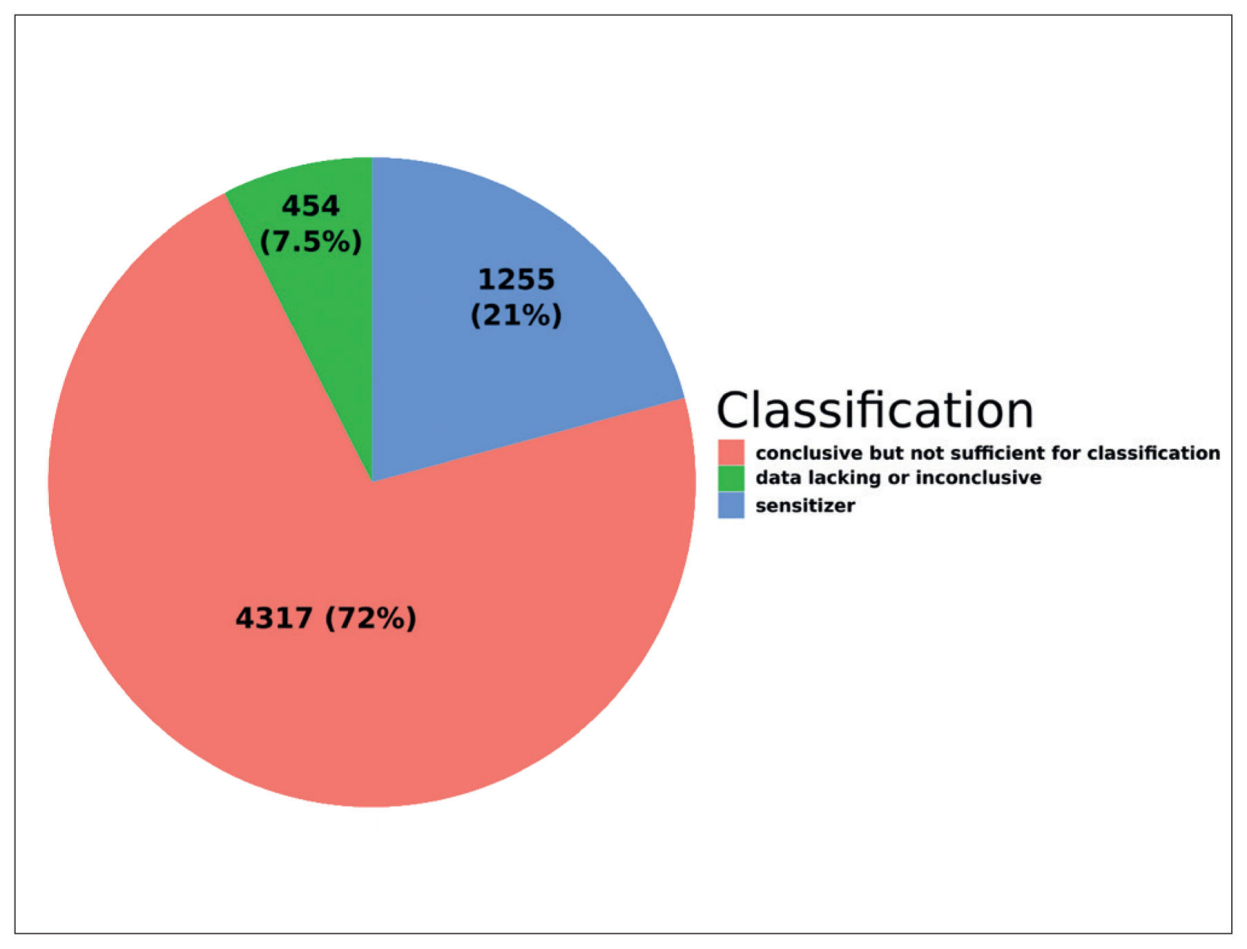
Prevalence of positives, “conclusive but not sufficient for classification” and “data lacking” or “inconclusive” outcomes for 6,026 ECHA dossiers with GHS Classification and Labeling data for H317 (may cause an allergic skin reaction) from REACH registrations 2008–2014

**Fig. 2:**
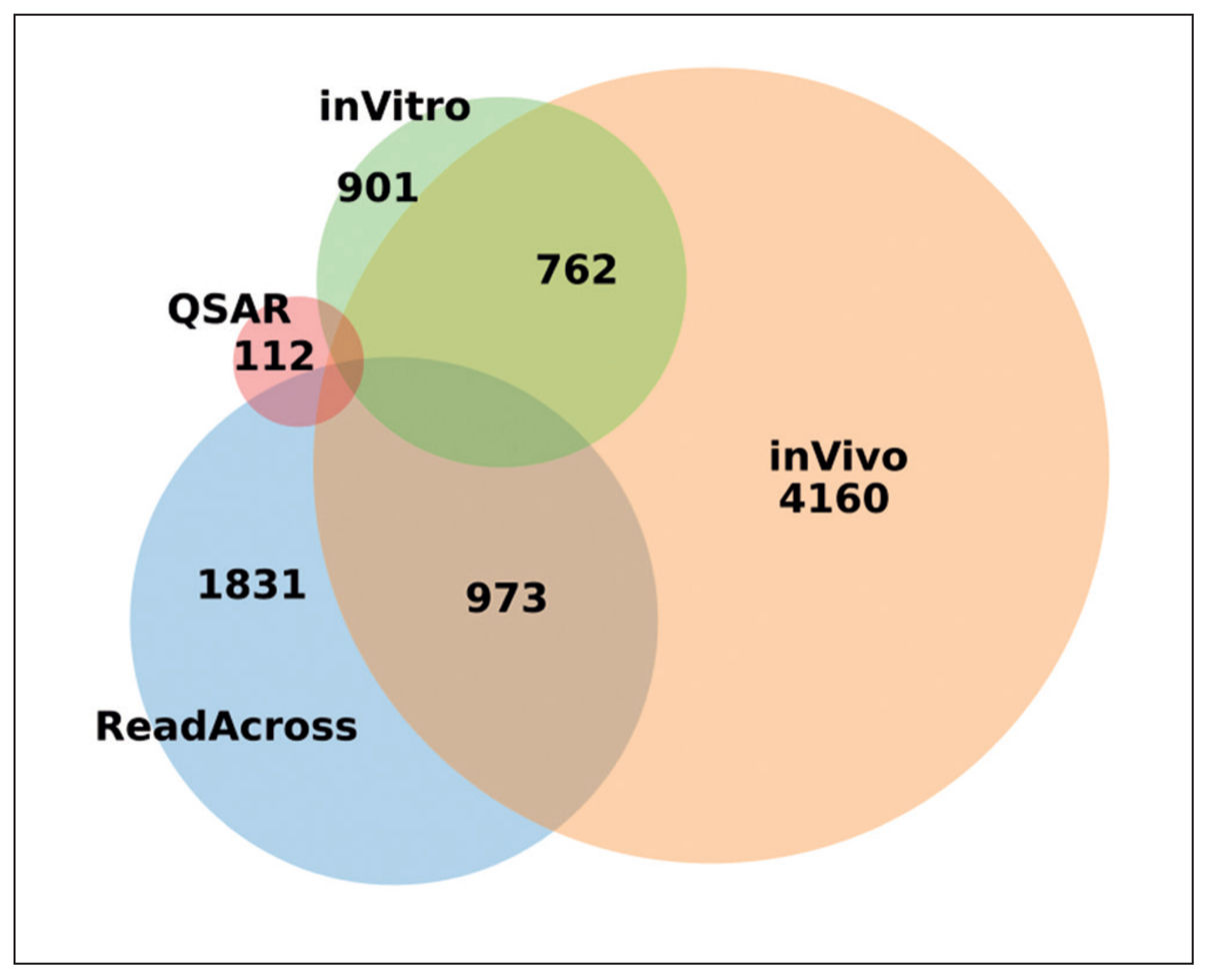
Diverse types of skin sensitization data on 8,739 REACH-registered substances from 2008–2014 Diagram shows number of substances with each type of study.

We extracted the prevalence of GHS hazards reported in substance dossiers. 21% of substances are identified as sensitizers by submitters via H317, “May cause allergic skin sensitisation”. Figure 1 shows the frequency of other H317 labels. Figure 2 gives the number of substances with sensitization data from either *in vitro*, *in vivo*, *read-across* or *QSAR* studies (noteworthy, 762 unique substances have both *in vivo* and *in vitro* data). Study types were assigned using mappings from study guidelines and language heuristics. We selected all studies meeting the below criteria:

1. Type contained the word “skin”, i.e., “exp key skin sensitization”.
2. Study contains “results and discussion” section.
3. Study contains “materials and methods” section.
4. Klimisch score less than or equal to 2.

The Klimisch score developed by OECD characterizes the reliability of the study based on defined criteria (Klimisch et al., 1997).

**Fig. 3:**
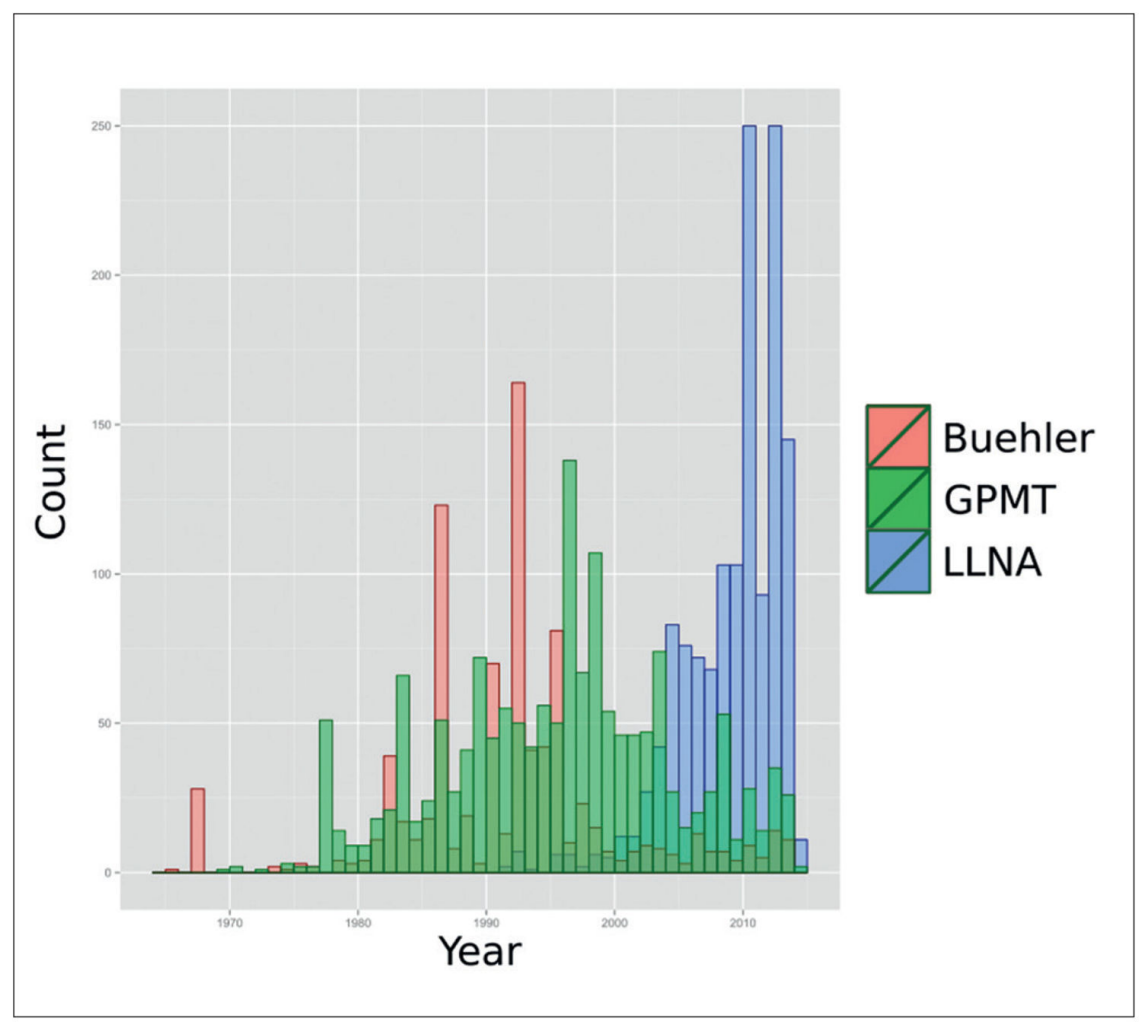
Histogram of study counts by date of execution of REACH-registered studies 2008–2014 Buehler and GPMT tests are most prevalent before 2000 and mouse LLNA are more prevalent after 2005.

**Fig. 4:**
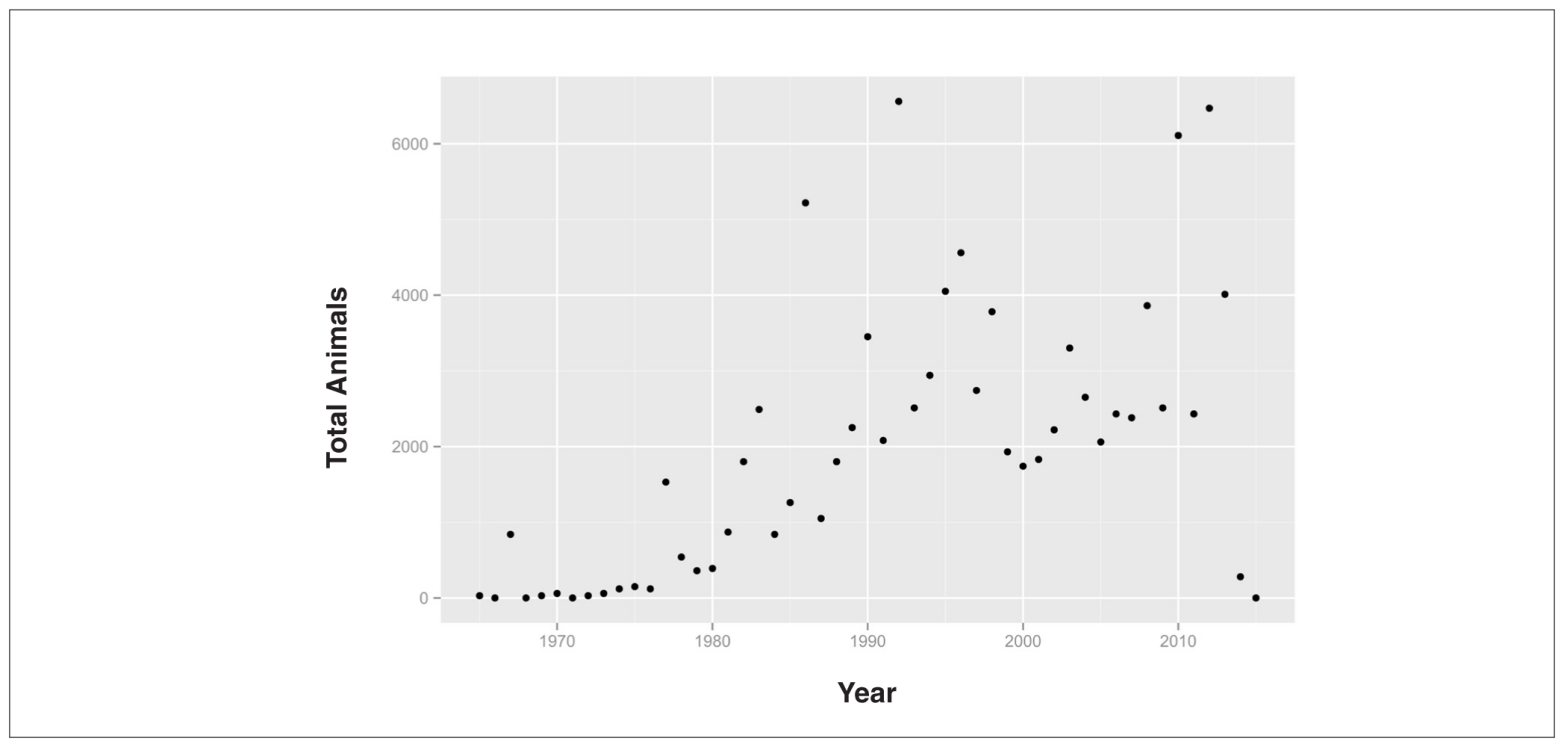
Estimate of number of animals used for experimental key skin sensitization studies since 1965 from REACH registration dossiers 2008–2014 Average animal numbers for each study type are used to calculate these numbers.

Use of the various guidelines for sensitization testing over time was visualized by analysis of reference dates and study types for sensitization studies. Figure 3 shows the counts by year of all experimental key skin sensitization studies of study type Buehler, GPMT or LLNA since 1970. Notably, the LLNA was validated in 1999 and is since then the preferred method in the EU. In spite of the increase in study counts during the progression to LLNA testing, Figure 4 shows only small changes in the estimated number of animals used for this increased testing. This lack of increases in estimated animal usage despite increased testing is likely due to the requirement of fewer animals for LLNA tests.

To evaluate the intra- and inter-reproducibility of skin sensitization tests, we analyzed all substances with two or more sensitization studies having an interpretation of “sensitizing” or “not sensitizing”, study type of “Guinea Pig Maximisation Test (GPMT)”, “Mouse Local Lymph Node Assay (LLNA)”, “Patch-Test” or “Buehler test”. We found 1,462 substances with multiple sensitization studies matching these constraints. Table 1 shows the results of that reproducibility analysis. It is important to note that the datasets used to evaluate the reproducibility between tests do not contain the same substances and for this reason percentage agreement should not be considered a direct comparison. All together, reproducibility ranged from 77% to 95% with 89% for the LLNA to reproduce itself. This means we can expect no alternative method to be better than this in direct comparisons if used on the same sets of substances.

LLNA (OECD TG 429) and GPMT (OECD TG 406) are frequently used to develop and test strategies of skin sensitization testing (Alvarez-Hamelin et al., 2005; Wuchty and Almaas, 2005; Newman, 2004). The extracted REACH data for *in vivo* skin sensitization contains a subset of 1,470 substances with mouse LLNA guideline studies and 2,787 substances with GPMT data, which is valuable for this purpose.

Recent datasets used for sensitization model evaluation and construction lack the number of substances shown in the REACH extraction here (Tab. 2). E.g., TIMES-SS, frequently cited and leveraged for its predictive strength, originally tested only 96 substances and was trained using 740 (Dimitrov et al., 2005).

### 3.2 Chemical similarity

**Fig. 5:**
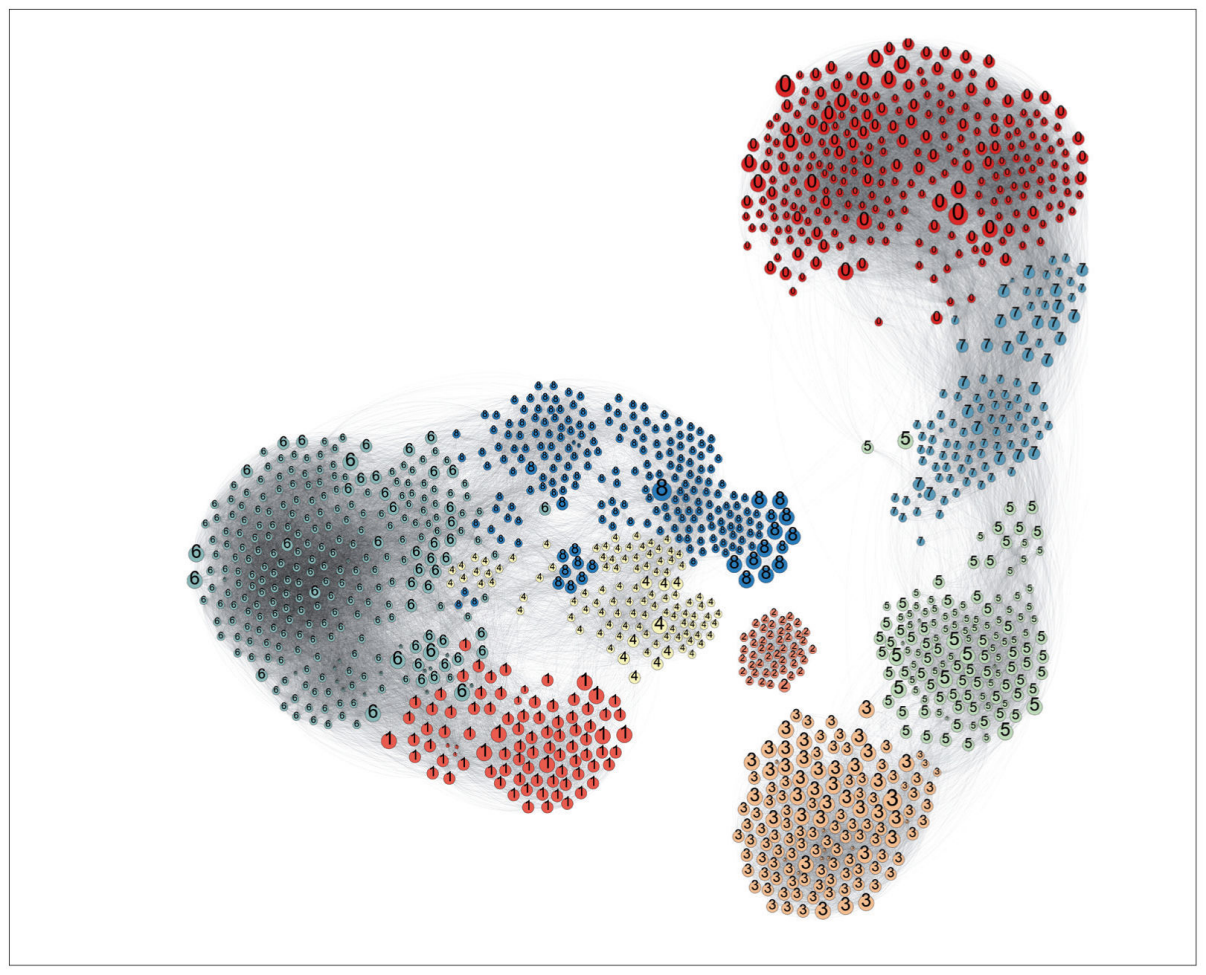
Chemical similarity map built from 3,116 chemicals with skin sensitization studies and mappings from REACH (registered 2008–2014) to PubChem K-Core filtrations was performed with K = 30. Nodes are colored by module (as determined by Blondel et al. (2008) algorithm). Numbers correspond to modules. Larger nodes are positive for more GHS health hazards (H300–H399). Edges are determined by chemical similarity and drawn between nodes with similarity ≥ 0.65.

A chemical similarity network was built from the 4,160 substances with *in vivo* skin sensitization data. We used PubChem’s 2D Tanimoto similarity metric, which required that we first mapped REACH substances to PubChem (Bolton et al., 2008). The resulting network displayed in Figure 5 is characterized by nodes representing substances. Each node size is determined by the number of ToxTree structural alerts. Edges are drawn between chemicals with similarity ≥ 0.65 with darker edges representing higher similarity.

The large size of the REACH extraction has a network effect on chemical prediction: As the reference dataset grows, the probability of neighbors existing for a new chemical increases. Jaworska and Nikolova-Jeliazkova’s (2007) paper “How can structural similarity analysis help in category formation?” used a 211 chemical dataset to evaluate several chemical similarity approaches. Our analyses are based on 3,116 chemicals with mappings to PubChem and skin sensitization data.

#### 3.2.1 PubChem 2D

Figure 5 shows a diverse chemical dataset along with some clear indication of clusters. To better visualize the modularity of this network, we used the K-Core filtration approach (Alvarez-Hamelin et al., 2005). Chemical modules, albeit dependent on chosen similarity metric, may make strong candidates for the evaluation of domains of applicability (Jaworska and Nikolova-Jeliazkova, 2007). Analysis of biological activity cliffs and other biochemical features as functions of modularity may also yield toxicological value.

**Fig. 6:**
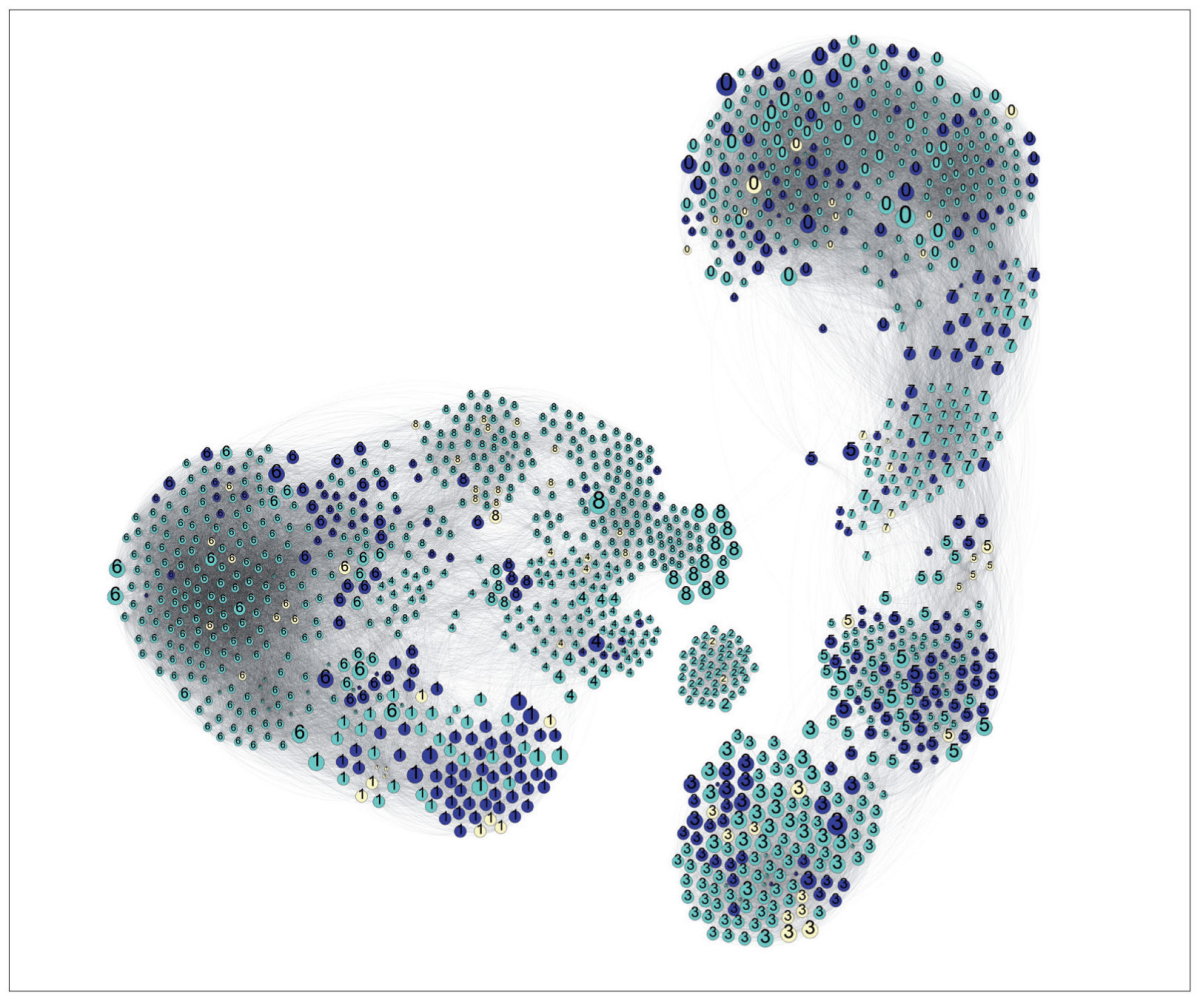
Chemical similarity network with experimentally classified sensitizers from REACH (registered 2008–2014) mapped to PubChem Sensitizers (dark blue) and non-sensitizers (turquoise) as well as unknowns in yellow are shown. Nodes are chemicals and edges are drawn between chemicals with > 65% similarity. Numbers indicate the module.

Leveraging the similarity network for skin sensitization analysis requires annotating nodes by their sensitization status. Figure 6 displays skin sensitizers in dark blue, non-sensitizers in turquoise, and unknowns (due to failed extraction or lacking data) in yellow. Visual inspection shows some modules lacking sensitizers (notably module 2), while others have high prevalence of hazard (module 1).

#### 3.2.2 Similarity subgraph analysis

Large datasets made possible via REACH extraction greatly enhance data exploration. Network visualizations allow for the discovery of important predictive features. Skin sensitization is frequently described as a function of electrophilicity and many models rely on features attempting to capture reactivity mechanisms. Given large enough reference data and clustering methods, experts may deduce reasonable sensitization mechanisms via analysis of interesting subgraphs.

Similarity subgraphs can be created and annotated automatically to aid in expert chemical evaluation, but rely on access to large datasets with endpoint information. Previously, software aiding in chemical screening processes have found use in subcategorizing Michael’s acceptors (Schultz et al., 2007, 2009).

**Fig. 7:**
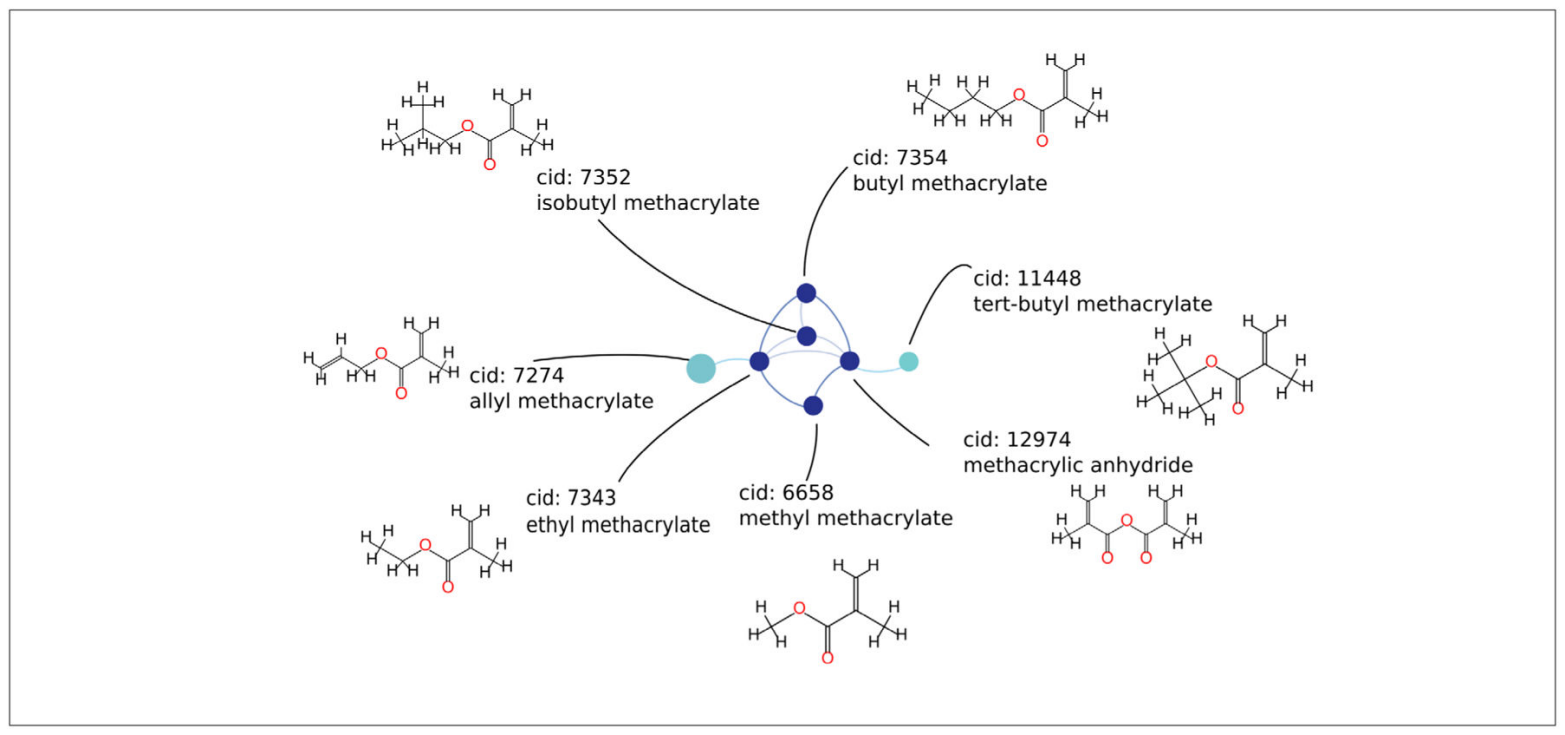
Module 1 similarity subgraph with methacrylate Michael’s acceptors of Figure 6 Edges drawn for substructure similarity > 90%. Dark blue signifies a skin sensitizer, light blue a non-sensitizer.

Figure 7 shows a subgraph of module 1 with highly similar (≥ 90%) methacrylates. Such subgraphs, particularly when made interactive, can help experts to elucidate mechanistic explanations of sensitization. Identification of differences between tert-butyl methacrylate and methacrylic anhydride could help discover mechanisms of reactivity within this subdomain. Alternatively, similarities between tert-butyl methacrylate and allyl methacrylate not shared by other chemicals in the subgraph could also be automatically explored.

### 3.3 Heuristic analyses

Modularity can be used to analyze domains of applicability. We developed a simple sensitization model using molecular weight and five ToxTree structural alerts (Enoch et al., 2007) and depict the number of structural alerts associated with a chemical by chemical node size where larger nodes have more structural alerts. To find ToxTree alerts, we used the ToxTree Maven repository^5^. These alerts capture electrophilic mechanistic information and include “Michael-type addition reaction”, “Schiff base formation”, “acylation”, “nucleophilic aromatic substitution” (SNAr) and “second order nucleophilic aliphatic substitution” (SN2). The final alert “Reactivity domain alert” is true if any other alert is true (Enoch et al., 2007).

The simple heuristic model follows the below pseudocode (alerts refer to the number of structural alerts for a chemical, and MW refers to chemical molecular weight in Da):

Equation 3 Heuristic sensitization algorithm using ToxTree structural alerts and MW

$$\begin{array}{c} \text{if}((\text{alerts}>0)\text{AND}\;(\text{MW}<500):\text{predict}\;\text{chemical}\;\text{as}\;\text{sensitizer}\\ \text{else}:\text{predict}\;\text{chemical}\;\text{as}\;\text{non}-\text{sensitizing} \end{array}$$

#### 3.3.1 Accuracy

**Fig. 8:**
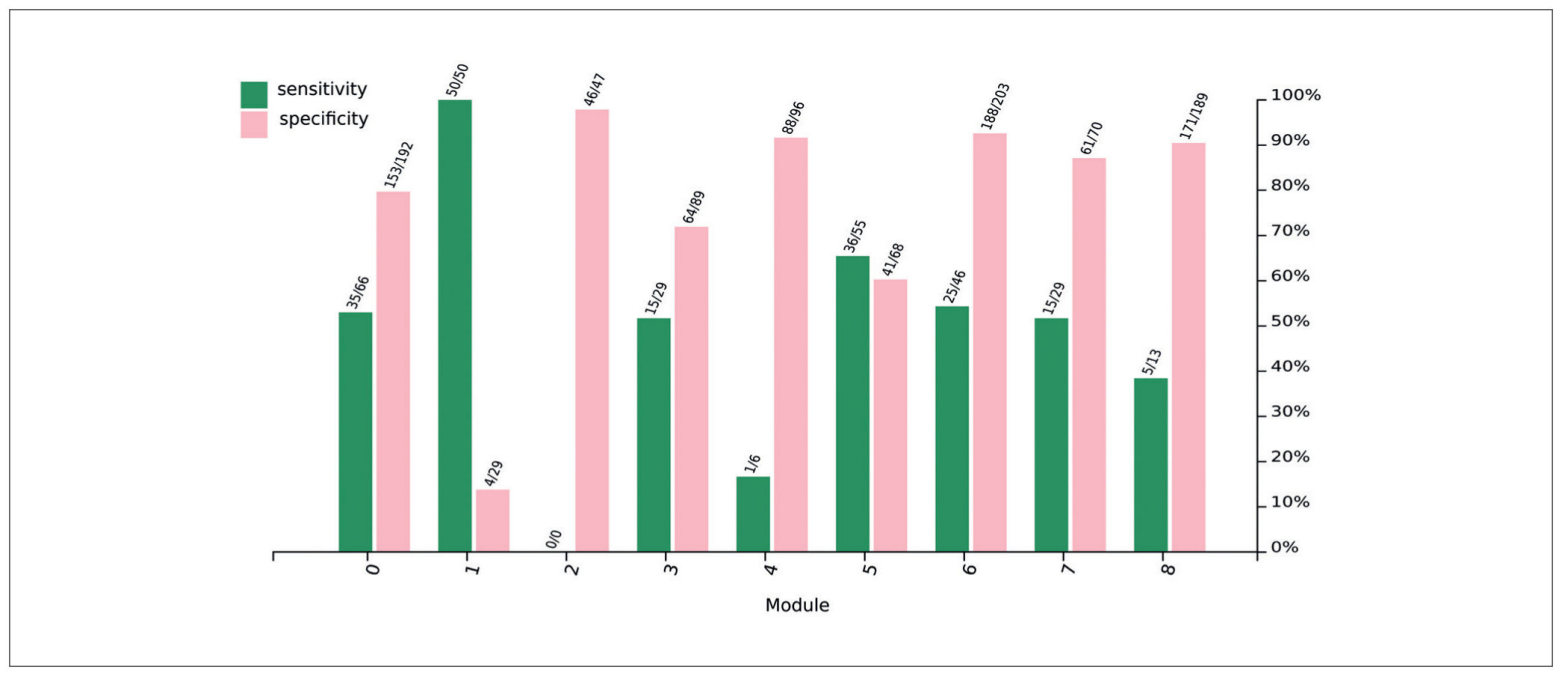
Sensitivity and specificity for ToxTree plus molecular weight sensitization heuristic defined on modules from Figure 5 A simple sensitization model using molecular weight and five ToxTree structural alerts (Enoch et al., 2007) was created: A substance was predicted positive if the molecular weight was below 500 and at least one alert (from the ToxTree Maven repository capturing electrophilic mechanistic information including “Michael-type addition reaction”, “Schiff base formation”, “acylation”, “nucleophilic aromatic substitution” (SNAr) and “second order nucleophilic aliphatic substitution” (SN2) as well as a final alert “reactivity domain alert” if any other alert is present) was found.

The ToxTree heuristic module specificity and sensitivity is displayed in Figure 8. With the exception of module 1, all modules show specificities over 60%. Module sensitivities are in the range of 10% to 65% with the exception of module 1 with sensitivity 100%. Over all 3,024 chemicals mapped from REACH to PubChem with skin sensitization data, the heuristic has a balanced accuracy of 65.8% with a specificity of 80.4% and sensitivity of 51.4%.

High specificities indicate that the absence of structural alerts effectively reduces the probability of sensitization. Lower sensitivities show that structural alerts do not necessitate sensitization. Module accuracy can be used to determine model applicability domains.

#### 3.3.2 Sensitization structural alerts

Structural alerts for skin sensitization identify substructures predictive for substance reactivity and sensitization proclivity. Distribution of structural alerts by module revealed wide variation (Tab. 3). Approximately 31% of mapped chemicals have the Michael acceptor alert and contain a,b-unsaturated ester, ketone or aldehyde functions. a,b-Unsaturated alcohols can also react as Michael acceptors after the alcohol group is oxidized to an aldehyde (Karlberg et al., 2013). Module 2 shows remarkably low reactivity with only one Schiff base alert (betaine), which represents the only misclassified substance in the module. Modules 1 and 3 have a large prevalence of Michael’s acceptors (> 80%) and module 3 has the greatest prevalence of acylation alerts.

Module characteristics can be further explored by examining the explanative power of structural alerts as a function of module. This analysis is done by examining, for each module, the information gain associated with each structural alert. Information gain is assessed by change in entropy.

Table 4 allows for comparison of structural alert information gain across modules by an entropy-normalized information gain metric (information gain divided by original entropy × 100). This normalized information gain takes on a value of 100 when an attribute perfectly separates a set of chemicals into sensitizers and non-sensitizers. It takes on a value of 0 when an attribute fails to reduce entropy in a set of chemicals. Lack of sensitizers in module 2 makes information gain in this module impossible.

Most notably, information gain due to structural alerts is surprisingly low in most modules. The reactivity domain (RD) attribute (positive when a chemical has any structural alert) is the most informative attribute for 4 out of the 9 modules, and also the most informative for the entire chemical space. The low overall information gain was attributed to a high incidence of false-positives associated with the RD alert.

In module 6 the presence of any reactivity domain yields high information gain. Since the heuristic algorithm, Equation 3, includes a split on reactivity alert, this high information yield explains the relatively strong accuracy seen for the heuristic in module 6 (Fig. 8). Of additional note is the strength of the acylation alert in module 6, which is higher than for any other module. It is possible that some frequent chemical substructures in module 6 predispose acylating agents to a greater probability of skin sensitization.

Module 5 sees a higher Schiff base alert information gain than the information gain associated with RD. Since the RD alert is true when any other alert is true (and can thus be considered a precautionary alert), its lack of predictive power in this module relative to more specific alerts indicates the weakness of a precautionary approach. By designating chemicals with any reactivity alert as sensitizers, it is possible to obtain lower accuracy than by intelligent selection of those alerts more strongly associated with the endpoint in question. In the case of module 5, using only the Schiff base alert to predict skin sensitization would yield stronger results than combining it with other alerts.

The high information value of SN2 in module 4 is due to the presence of a single SN2-positive chemical, bisisobutyryl peroxide, and should be considered of insufficient power to draw conclusions. The scarcity of sensitizers in module 4 and 2 (6 sensitizers and 0) limited any conclusions being drawn from information gain.

The low prevalence of SNAr in this chemical dataset (only 20 instances) makes any meaningful information gain highly unlikely for this attribute. In and of itself low prevalence does not disqualify SNAr from being of strong predictive power, however, if we analyze all chemicals with a positive SNAr alert we see that 7 are considered sensitizers and 13 are non-sensitizers. This means that the SNAr alert does a poor job (albeit on a small sample) of separating these two classes.

Despite its high frequency in many modules, the Michael’s acceptor alert does not provide much information for skin sensitization. Subcategorization of Michael’s acceptors can improve the predictive capacity of this alert. Schultz et al. (2009) show that further categorization can influence reactivity of Michael’s acceptors. Identification of the subcategorizing substructures may be aided by generation of interactive subgraphs built on our existing code base; Figure 7 shows a preliminary example.

#### 3.3.3 Molecular weight

Molecular weight is an ubiquitous parameter used within skin sensitization prediction models. In our heuristic Equation 3, we use the 500 Da molecular weight rule, which stipulates that substances over 500 Da are non-sensitizers (Bos and Meinardi, 2000). In 2013, Roberts et al. (2013) determined the 500 Da cut-off to be a “myth”, identifying 5 out of 13 substances with a molecular weight > 500 Da (out of a 700 chemical dataset) to be sensitizers (Roberts et al., 2013). We corroborate Roberts’ results with REACH evidence of 49 sensitizing chemicals with a molecular weight greater than 500 Da.

The low information gain associated with molecular weight prompted the use of a binary search for the molecular weight threshold yielding greatest information gain per module. Rather than setting a threshold beyond which we expect no skin sensitizers, we simply desire a threshold that optimally separates sensitizers and non-sensitizers and yields the greatest reduction in Shannon entropy (see Section 2.7). This approach yields a molecular weight threshold of 309 for all chemicals with ranges from 110 to 842 for individual modules.

There is an equal number of sensitizers above and below a molecular weight threshold of approximately 200 Da (305 below, 318 above, data not shown). There are only 100 sensitizers with a molecular weight greater than 377 and only 23 sensitizers with a molecular weight greater than 644 Da. However, total substance count also falls. The ratio of sensitizers to total chemicals gradually falls as molecular weight increases (data not shown). The low information attributed to molecular weight in Table 4 and the gradual reduction in relative sensitization count as the molecular weight threshold is increased suggests that, rather than some molecular weight threshold making skin penetration unlikely, molecular weight increases may simply reduce the number of reactive molecules as suggested by Roberts et al. (2013).

### 3.4 K-nearest neighbors classification

Our KNN variant classifies chemicals by finding all neighbors with similarity greater than a parameter *T*. The majority label of neighbors is then used to predict the class of the chemical in question.

Accuracy metrics for different values of *T* are given in Table 5. We find this simple algorithm outperforms our heuristic approach already at the 75% minimum-similarity threshold, whereby chemicals are considered neighbors if their substructure similarity is greater than or equal to 75%. At higher similarity thresholds the balanced accuracy increases. Lower similarity thresholds decrease sensitivity faster than specificity.

The surprising strength of this naïve similarity based classification algorithm is impacted by several factors. The REACH extraction provides a large dataset, which improves the probability of neighbor existence for any given chemical (this is a network effect). A smaller dataset would result in poorer accuracy due to insufficient neighbors at large similarity thresholds. The success of this approach indicates that the PubChem 2D substructure features must bear some predictive strength for skin sensitization. Substructure information is related to chemical features such as electrophilicity and reactivity.

Figure 7 shows a small subgraph of Michael’s acceptors with high similarities (90%). The identification of this small subgraph of highly similar chemicals suggests that similarity graph analysis could be used by experts for identifying locally predictive chemical characteristics. One might use this subgraph to try and explain why allyl methacrylate and tert-butyl methacrylate are non-sensitizers while the remaining methacrylates are sensitizers.

## 4 Discussion

This analysis of skin sensitization data for industrial chemicals registered in REACH shows the wealth of information that can be used to tailor and optimize future testing strategies. Our preliminary analysis shows how such a large dataset can be leveraged. This can considerably reduce testing needs and related costs (Hartung and Rovida, 2009; Rovida and Hartung, 2009). Computational approaches benefit enormously from the amount and quality of the data. Already relatively simple algorithms can make reasonable predictions, also supporting the concept of read-across (Patlewicz et al., 2014). Noteworthy, the majority of new tools and testing strategies were developed over the last decade on the same set of only about 145 substances (Natsch et al., 2013). In a data-rich environment, conclusions can be drawn reasonably from neighboring substances, especially when structural properties are backed with biological profiling (Zhu et al., 2016, this issue).

The variability of the LLNA was pointed out by Urbisch et al. (2015): By retesting 22 LLNA performance standards in the standard LLNA protocol, a reproducibility of only 77% was found (Kolle et al., 2013). Recently, Hoffmann (2015) analyzed the variability of the LLNA test, using the NICEATM database. Repeat experiments for more than 60 substances were analyzed in terms of skin sensitization potential, i.e., discriminating sensitizers from non-sensitizers: The false positive rate ranged from 14–20% (false negative rate 4–5%). In terms of skin sensitization potency, the chance of assigning a substance to the next higher or next lower potency class was approximately 10–15%. Here, similar results were found for a much larger substance set of 1,462 substances that had undergone multiple sensitization studies.

Michael acceptors, substances accepting nucleophilic substitutions, are suggested to show higher hapten formation by binding to proteins and thus higher skin sensitization potential; they were also predicted as sensitizers with the highest accuracies by *in vitro* tools (Urbisch et al., 2015). It is thus astonishing that the Michaels acceptor alert gave little information value for the identification of sensitizers. It is possible that the Michael’s acceptor alert has a strongly limited domain of applicability, or even that it is generally non-informative. The overestimation of the relevance of this attribute in prior models may be a consequence of the small datasets used for model construction.

Strong balanced accuracies across this large dataset using a simple KNN algorithm provides evidence for the promise of more advanced similarity approaches on the same dataset. In addition to algorithmic analysis, one could envision combining advanced similarity-based classification techniques with graph visualizations to aid experts in identifying subgraph-specific substructures predictive of biological activity cliffs.

### 4.1 Chemical similarity

This similarity approach serves as a proof of principle and is subject to several flaws. The similarity paradox described by Martin et al. (2002), whereby small changes in chemical structure lead to large biological changes, is not handled in this approach. Additionally, large molecules have more substructures and therefore a greater probability of similar neighbors. Many substructures are redundant with each other, for instance the fourth feature in the PubChem 2D conformational fingerprint is “≥ 32 H”. If this substructure is present then the first three substructures all must be present (≥ 4 H, ≥ 8 H, ≥ 16H).

More advanced similarity techniques employ approaches that account for metabolism, group chemicals into reactivity domains, use supervised learning algorithms to tailor similarity metrics for the endpoint in question and more (Nikolova and Jaworska, 2003). Jaworska and Nikolova-Jeliazkova (2007) show strong alternatives for similarity metrics (albeit on a smaller dataset). Self-organizing maps have notably been used to make context-specific similarity metrics. Kleinstreuer et al. (2014) use this approach for phenotypic screening of the Tox-Cast chemical library.

We pursued a naïve approach here with the intention to show that by simply increasing the size of the dataset we can create stronger models. Future research with more advanced approaches could improve the performance.

### 4.2 QSAR

Sensitization model performance varies by applicability domain. In their evaluation of Times-SS, Patlewicz et al. (2007) distinguish three categories of models: Local models make the definition of this domain explicit as they are typically defined on a single chemical class. Global models defined on a single mechanism of action may also have a well-defined applicability domain limited to chemicals for which the given mechanism can be confidently determined. Universal models are based on sensitization datasets comprising various chemistries and multiple mechanisms. All of these models benefit from larger datasets in training and evaluation, with the greatest benefits realized by computational models unable to use knowledge not directly present in the data. (Q)SARs benefit from larger datasets through improved training and strengthened definition of the applicability domain. Whereas expert models can explicitly define applicability domains through structural rules, (Q)SAR rely on implicit domains defined by the scope of training sets (Netzeva et al., 2005).

Extraction of skin sensitization data from REACH is currently a nontrivial task, and the substance mapping performed here may be error-prone and incomplete. Modern skin sensitization models typically predict reactivity levels (EC_3_ values for LLNA) or discretizations thereof. While it is possible to extract EC_3_ values from REACH, the variability in the means of entering EC_3_ data into chemical dossiers makes it difficult to accurately extract the required information. The data extracted for these analyses were mapped from natural language to numeric or categorical values, which will have a certain intrinsic error that could be avoided by more structured data and formal data entry protocols.

## 5 Conclusions

The characterization of the REACH chemical universe (at the time of extraction, 2014) in the context of skin sensitization showed a proportion of 21% sensitizers among these predominantly high-production volume chemicals. The large number of repeat studies and the overlap of methods used for assessment of many chemicals allowed the investigation of the reproducibility of the *in vivo* methods. This showed a range of 80–90% and therefore no alternative method or integrated testing strategy (ITS) should be expected to perform better than this as long as we use these tests as points of reference.

The extracted database presents the largest repository of *in vivo* skin sensitization data and indicates ECHA as a still larger repository of sensitization and other animal testing results. This will be most valuable when now exploring new tests/ITS and *in silico* approaches.

With improved data structure and machine-readable data, the ECHA datasets could transform sensitization modeling. In this publication, several obvious extensions could be made with improved data. The prediction of a binary outcome (sensitizer vs. non-sensitizer) in this article was necessitated by failing to extract potency information where available in ECHA dossiers. The available *in vitro* data in the database have not been analyzed and exploited yet. The promising predictivity of rather naïve prediction models from chemical neighbors suggests that such advanced predictions could actually bring predictions into the range of *in vivo* reproducibility.

One goal of this publication is to underscore the importance of data availability and structuring data in a machine-readable format – while REACH in many ways has a workable ontology for classifying endpoints, much could be improved by more formal data structures for results extracted from the main guideline-compliant studies. This would make this huge investment into consumer safety also an investment into the future of safety sciences.

Furthermore, it is our hope that our arguments and referenced articles will motivate the publication of REACH data in the scientific community and to inform the general public. An open REACH platform would allow third parties to investigate concepts such as testing redundancies and hazard distributions, and could accelerate much other toxicological research. As we have demonstrated, REACH does already, but could even more widely, provide computational toxicology with an unparalleled dataset.

## Figures and Tables

**Tab. 1:** Classification agreement on chemicals with at least two sensitization studies in REACH dossiers from 2008–2014 Studies found by searching for all studies with *studytype* = Buehler, GPMT, Patch-Test or LLNA.

	Buehler	GPMT	Patch-test	LLNA
**Buehler**	95.1% (344 chem.)	91.8% (364 chem.)	87.8% (58 chem.)	76.8% (212 chem.)
**GPMT**		93% (624 chem.)	90.5% (107 chem.)	77.4% (403 chem.)
**Patch-test**			92.1% (24 chem.)	78.3% (40 chem.)
**LLNA**				88.5% (296 chem.)

**Tab. 2:** Model datasets derived from LLNA and GPMT data These datasets were used to build or evaluate skin sensitization models. The table lists 1) bibliographic source or model (with citation) 2) Use of dataset (used to build a model, evaluate models or both) 3) number of substances in dataset.

Article or Model	Use	Substances
**Toxtree** (Enoch et al., 2007)	Build/Eval	210
**DEREK** (Barrat and Langowski, 1999)	Build/Eval	85
**TIMES-SS** (Dimitrov et al., 2005)	Build	740
**TIMES-SS** (Dimitrov et al., 2005)	Eval	96
**Computers versus Reality** (Teubner et al., 2013)	Evaluation	100
**Times-SS** (Patlewicz et al., 2007)	Evaluation	40
**Reach extraction skin *in vivo*** (this study)	Build/Eval	4160

**Tab 3:** Prevalence of structural alerts by module (modules in Fig. 5) Column maximums in blue. SN2 = Second order nucleophilic aliphatic substitution. MA = Michael-type addition reaction. SB= Schiff base alert. AA = Acylating agent. SNAr = Nucleophilic aromatic substitution. RD = Reactivity domain alert (true when any other alert is true). Diverse prevalences indicate unique distributions of reactivity alerts by chemical module.

Module	SN2	MA	SB	AA	SNAr	RD	Count
**ALL**	5.3%	13.1%	7.6%	9.1%	0.7%	30.9%	3024
**Undefined**	5.9%	7.2%	9.2%	8.0%	0.9%	27.9%	1509
**0**	10.0%	13.5%	4.6%	12.0%	0.7%	34.1%	258
**1**	8.8%	89.8%	1.2%	7.5%	0.0%	97.4%	79
**2**	0.0%	0.0%	2.1%	0.0%	0.0%	2.1%	47
**3**	5.0%	81.3%	0.0%	37.2%	1.6%	96.6%	118
**4**	0.9%	0.0%	0.0%	8.8%	0.0%	8.8%	102
**5**	4.8%	23.5%	17.0%	17.0%	0.0%	56.1%	123
**6**	4.4%	0.0%	6.0%	10.8%	0.0%	18.4%	249
**7**	2.0%	15.1%	6.0%	3.0%	0.0%	26.2%	99
**8**	0.5%	9.9%	6.9%	0.5%	0.0%	11.3%	202

**Tab. 4:** Structural alert and molecular weight information by module Comparison of structural alert information gain across modules by an entropy normalized information gain metric (information gain divided by original entropy × 100). This normalized information gain takes on a value of 100 when an attribute perfectly separates a set of chemicals into sensitizers and non-sensitizers. It takes on a value of 0 when an attribute fails to reduce entropy in a set of chemicals. Lack of sensitizers in module 2 makes information gain in this module impossible. Row maximums in green. Column maximums in pink. Cells that are both a row and column maximum in orange.

Module	Entropy	SN2	MA	SB	AA	SNAr	RD	MW	MW Threshold
**ALL**	0.72	0.61	3.76	1.53	0.17	0.08	6.61	0.73	309
**None**	0.69	0.09	1.76	1.51	0.02	0.09	2.54	1.20	306
**0**	0.80	4.48	3.43	1.27	0.00	0.38	7.06	3.32	439
**1**	0.98	0.62	3.42	0.93	1.38	0.00	3.04	15.85	329
**2**	0.00	na	na	na	Na	na	na	na	na
**3**	0.78	0.27	4.80	0.00	0.43	4.41	1.55	6.76	682
**4**	0.31	12.82	0.00	0.00	0.96	0.00	0.96	10.83	174
**5**	0.98	0.37	0.53	9.84	2.10	0.00	5.36	3.20	110
**6**	0.68	1.94	0.00	1.54	15.25	0.00	20.70	1.87	842
**7**	0.86	0.35	6.48	3.35	0.03	0.00	11.44	8.99	121
**8**	0.33	5.80	5.31	1.83	0.25	0.00	7.32	7.88	173

**Tab. 5:** Accuracy metrics for KNN variant given different minimal-similarity parameters For a given chemical neighbors used for prediction are constrained to those with similarity ≥ the minimum similarity. Predictions are made for all chemicals with one or more neighbors.

Min. Similarity	Chemicals	TP	TN	FN	FP	Sensitivity	Specificity	BAC	Accuracy
0.95	525	52	429	20	24	0.68	0.96	90.82	0.92
0.9	1189	142	870	85	92	0.61	0.91	0.76	0.85
0.85	1738	220	1238	130	150	0.59	0.90	0.75	0.84
0.75	2288	224	1616	170	278	0.45	0.90	0.68	0.80

## Footnotes

1 Bureau of Labor Statistics: Industry and illness data. Available online at: http://www.bls.gov

2 https://www.mongodb.org/

4 PubChem Substructure Fingerprint v1.3 (2009), http://pubchem.ncbi.nlm.nih.gov

5 http://toxtree.sourceforge.net/skinsensitisation.html
